# Willingness, Challenges, and Perceptions of Patients’ Family Members and Health Care Providers Regarding Virtual Reality–Based Preoperative Intensive Care Unit Visits for Elective Surgery: Qualitative Study

**DOI:** 10.2196/84418

**Published:** 2026-08-10

**Authors:** Chunmei Fan, QI Wang, Jicheng Zhang, Zhenfeng Zhou, Zhengang Wei, Congcong Liu, Min Ding

**Affiliations:** 1 Intensive Care Unit, Shandong Provincial Hospital Affiliated to Shandong First Medical University, Second Floor of Ward One, Jinan, Shandong, China, 86 1-786-297-1621

**Keywords:** virtual reality, preoperative visit, intensive care unit, patient education, qualitative research

## Abstract

**Background:**

Perioperative information asymmetry regarding intensive care unit (ICU) care remains a critical unmet clinical need. Traditional preoperative visits focus primarily on surgical risks but provide insufficient education about postoperative ICU stays, leading to widespread cognitive biases and anxiety among patients’ family members. Virtual reality (VR) technology offers immersive, standardized information delivery that could address these gaps, yet research on VR-based preoperative ICU visits from the dual perspectives of health care providers and patients’ family members remains limited.

**Objective:**

This study aimed to explore the willingness, perceptions, and information needs of ICU health care providers and patients’ family members regarding VR-based preoperative ICU visits for patients undergoing elective surgery, and to identify core user-centered design requirements and implementation barriers to inform evidence-based system development.

**Methods:**

A descriptive qualitative study was conducted in 2 ICUs of a tertiary hospital in Jinan, Shandong Province, China, between April and July 2025. Twenty-nine participants (n=15 nurses, n=6 physicians, and n=8 patients’ family members) were recruited using purposive sampling with maximum variation. The data were collected via semistructured face-to-face interviews and analyzed using Braun and Clarke’s 6-phase reflexive thematic analysis framework, rooted in a constructivist epistemology. This study adhered to the COREQ (Consolidated Criteria for Reporting Qualitative Research) guidelines.

**Results:**

A total of 29 participants were interviewed (including n=15 nurses, n=6 physicians, and n=8 patients’ family members), leading to the identification of four main themes: (1) patients’ family members’ perceptions and unmet information needs regarding the ICU, (2) willingness and attitudes toward VR-based preoperative ICU visits, (3) user-centered design requirements for VR systems, and (4) anticipated implementation barriers.

**Conclusions:**

This study provides the first dual-perspective needs assessment of VR-based preoperative ICU visits in China. All stakeholders expressed high anticipated acceptance of this technology, though their specific priorities differed. These findings establish a foundational evidence base for developing user-centered VR preoperative visit systems and support further evaluation of their clinical effectiveness and implementation feasibility.

## Introduction

Preoperative visits are a core component of perioperative care that facilitate structured information exchange between health care providers and key stakeholders [1-3]. In China, the rapid development of critical care medicine has resulted in an increasing proportion of surgical patients being transferred to the intensive care unit (ICU) postoperatively. However, patients’ family members typically have extremely limited prior understanding of the ICU, often forming negative stereotypes based on fragmented or distorted information from nonmedical sources. Traditional face-to-face verbal communication between ICU nurses and patients’ family members often fails to effectively convey complex ICU environments, care workflows, and medical equipment, leading to persistent information asymmetry between health care providers and families [4,5]. This not only causes significant psychological distress for families but also substantially increases communication burdens for already overstretched ICU health care providers [6-9]. Despite these well-documented challenges, research on preoperative ICU visits remains far less developed than operating room-based preoperative education [10].

Virtual reality (VR) technology, with its unique immersive, interactive, and standardized features, offers a promising solution to these long-standing communication barriers [5,11]. Unlike verbal descriptions or static images, VR can provide a realistic ICU environment and complete postoperative care processes, enabling consistent and comprehensive information delivery to all stakeholders. While VR has demonstrated effectiveness in various health care education settings, its application to preoperative ICU visits targeting patients’ family members and health care providers remains largely unexplored [12,13].

This study represents the first phase of developing a user-centered VR-based preoperative ICU visit system, focusing exclusively on the dual perspectives of patients’ family members and health care providers. Given the complexity of multistakeholder needs and journal length constraints, patient perspectives will be reported separately in a subsequent publication. Specifically, we aim to explore the willingness, perceptions, and information needs of ICU health care providers and patients’ family members regarding VR-based preoperative ICU visits and identify core design requirements and implementation barriers to inform evidence-based system development.

## Methods

### Overview

A descriptive qualitative research design was adopted. The data were collected through semistructured, face-to-face interviews. Given the limited existing knowledge regarding the application and perceptions of VR-based preoperative ICU visits, this design was chosen to capture the complexity of the phenomenon and obtain credible data that closely reflect participants’ genuine thoughts and experiences.

### Ethical Considerations

This qualitative study was conducted by a research team from the ICU of a tertiary grade A hospital in Jinan, Shandong Province, China, and was approved by the hospital’s Ethics Committee (approval number 2025‐929). Informed consent was obtained from all participants. All participants were informed that their participation was voluntary and that they could withdraw from the study at any time without any adverse consequences. To protect participants’ privacy, each participant was identified by a code instead of their real name during data analysis. Additionally, no financial compensation was provided to any participant. All original interview data and participant information were anonymized and stored on an encrypted cloud drive to ensure confidentiality.

### Study Setting and Participants

This study used purposive sampling with maximum variation to ensure the diversity of participant perspectives, which is appropriate for exploratory qualitative research aiming to understand a range of experiences and needs [14]. For health care providers, we intentionally selected participants with varying years of ICU experience (1‐30 y), professional titles (nurse-in-charge to chief nurse, attending to chief physician), and clinical roles (bedside nurses, charge nurses, intensivists) to capture diverse clinical perspectives. For patients’ family members, we recruited individuals across different age groups (26‐70 y), educational levels (primary school to master’s degree), and relationships to patients (spouse, child, parent) to reflect their inherent heterogeneity.

Based on these predefined criteria, potential participants were identified by a researcher with the charge nurses of 2 ICUs at the aforementioned hospital. Eligible individuals were approached in person between April and July 2025, provided with detailed study information, and invited to participate; 3 patients’ family members declined due to time constraints. All selection decisions were reviewed and approved by an ICU nursing expert with 10 years of clinical experience to ensure alignment with study aims.

All participants were fully informed of their rights regarding study participation and provided written informed consent. They were notified that they could withdraw consent at any time without providing a reason or facing any negative consequences. The inclusion criteria were as follows: for patients’ family members being an immediate relative of an ICU patient and fully involved in the patient’s treatment decision-making process; for health care providers: having at least 1 year of working experience in the ICU. The exclusion criterion was the inability of patients’ family members or health care providers to participate in the interview (eg, due to physical or cognitive limitations). To ensure comprehensive reporting, this study adheres to the COREQ (Consolidated Criteria for Reporting Qualitative Research) checklist [15].

### Data Collection

Guided by a descriptive qualitative research methodology, the data were collected via in-depth, semistructured interviews. Interviews were conducted face-to-face in either the conference room (for physicians and nurses) or the private conversation room (for patients’ family members) of the ICU. These settings were quiet, comfortable, and private, with no interruptions, to ensure the continuity of thought for both the researchers and participants. The researchers were graduate students who had completed qualitative research courses and possessed interview experience. An initial interview guide was developed following discussions within the research team and consultation with qualitative research experts. A pilot study was then conducted with 1 nurse, 1 physician, and 2 patients’ family members to identify and address potential issues. The final interview guide was finalized after revisions, with details presented in Textboxes 1 and 2. Before each interview, participants were provided with a detailed explanation of the study’s purpose, methods, and confidentiality principles, and they signed the informed consent form on-site. Participants were informed of the concept and purpose of VR-based preoperative ICU visits but did not experience or view an actual VR system or training video. During the interviews, the researchers listened attentively to participants’ responses, promptly asked follow-up questions to clarify ambiguous content, and ensured the completeness and accuracy of the information. Participants were encouraged to share their willingness, needs, and perceptions regarding the VR-based preoperative visit system, with minimal interference from the researchers’ personal views. All interviews were anonymously recorded using a voice recorder, and nonverbal behaviors (eg, facial expressions, body language) of participants were documented simultaneously. Each participant was interviewed once: interviews with patients’ family members lasted approximately 25 to 45 minutes, while those with health care providers lasted approximately 30 to 40 minutes.

Textbox 1. **Interview guide for patients’ family members.**

**Questions**

1. What is your level of understanding of the intensive care unit (ICU)? Through which channels do you obtain information related to the ICU?
2. Have you previously known about or used virtual reality (VR)–based systems or devices? If yes, what were the purposes and functions of your use?
3. What is your perspective on using VR technology for preoperative visits in the ICU? What functions do you hope such a system will have? In your opinion, which types of knowledge content and presentation methods are more likely to meet your needs?
4. What are your views on the feasibility and potential value of applying VR technology to preoperative ICU visits?
5. During the use of the VR-based preoperative visit system for the ICU, what obstacles do you think might be encountered? How can these obstacles be addressed?
6. Do you have any additional suggestions regarding the design and application of the VR-based preoperative visit system for the ICU?

Textbox 2. **Interview guide for intensive care unit (ICU) physicians and nurses.**

1. Have you heard of preoperative visits before? In your opinion, what are the main difficulties and challenges currently faced by preoperative visits?
2. Have you previously known about or used virtual reality (VR)–based systems or devices? If yes, what were the purposes and functions of your use?
3. From the perspective of clinical practice, what is your view on using VR technology for preoperative visits in the ICU? What functions do you hope such a system will have? In your opinion, which types of knowledge content and presentation methods are more likely to meet your needs?
4. What are your opinions on the feasibility and potential value of applying VR technology to preoperative ICU visits?
5. Are you willing to use a VR system for preoperative visits? Why or why not?
6. During the promotion of VR-based preoperative ICU visits, what obstacles do you think might be encountered? How can these obstacles be addressed?
7. Do you have any additional suggestions regarding the design and application of the VR-based preoperative visit system for the ICU?

### Data Analysis

Within 24 hours of each interview, the audio recordings were verbatim transcribed into text by the researchers themselves, and the transcribed textual data were returned to the participants for verification, thereby improving the credibility of the data. Subsequently, the transcribed text was imported into NVivo 14.0 software (Lumivero) for analysis, with this process conducted simultaneously and independently by the primary researcher and another master’s student trained in qualitative methods.

This study adopted Braun and Clarke’s 6-phase reflexive thematic analysis framework, which is rooted in a constructivist ontology and interpretive epistemology [16]. This approach posits that themes are actively constructed and interpreted by researchers through reflexive engagement with data, rather than passively discovered as pre-existing entities. The six analytical phases were implemented as follows: (1) familiarization with the data: both researchers independently read each transcript a minimum of 3 times, documenting detailed reflexive notes on initial impressions, recurring meaning patterns, and preliminary insights. This phase aimed to immerse researchers in the dataset and generate initial coding ideas. (2) Generating initial codes: codes were generated inductively directly from the data, unconstrained by preexisting theoretical frameworks or the researchers’ a priori assumptions. Each meaningful text segment (ranging from short phrases to full paragraphs) was assigned a descriptive code capturing its core meaning. A total of 103 initial codes were generated across all transcripts. (3) Searching for themes: similar codes were collated and grouped to construct potential themes, mapping how distinct codes combined to represent broader meaning patterns across the dataset. (4) Reviewing themes: the 2 researchers independently reviewed initial themes to confirm they coherently aligned with the full dataset and that supporting coded extracts were consistent with each theme’s core meaning. Discrepancies were resolved through discussion; a third qualitative research expert was consulted if consensus could not be reached. (5) Defining and naming themes: each theme was clearly delineated and named to capture its core conceptual meaning, with subthemes developed within each main theme to convey nuanced layers of insight. (6) Producing the report: the findings were synthesized into a coherent narrative supported by illustrative participant quotes.

Data collection proceeded separately for each stakeholder group (nurses, physicians, and patients’ family members) given their distinct roles and perspectives. Recruitment and data collection within each group continued until sufficient depth and richness of interpretive meaning were achieved, with no new distinct meaning patterns identified in the final 2 consecutive interviews per group [15]. One additional interview was conducted per group to confirm the sufficiency of the data’s interpretive depth [17].

### Researcher Characteristics and Reflexivity

This study was conducted by a nursing research team with expertise in critical care and qualitative methods. The interviewer was a woman master’s student in nursing with 3 years of ICU clinical experience and formal qualitative research training. None were directly involved in the clinical care of participants’ family members to minimize power imbalances. The corresponding author is an ICU nursing specialist with 10 years of clinical and research experience who supervised the entire study.

Reflexivity was maintained throughout the research process. Interviewers kept reflexive journals to document their preconceptions, and regular team debriefings were held to discuss emerging themes. Independent dual coding by 2 researchers was used to enhance analytical credibility, with discrepancies resolved through consensus discussions.

## Results

### Descriptive Results and Study Population

A total of 29 participants were interviewed between April and July 2025, including 15 ICU nurses, 6 ICU physicians, and 8 patients’ family members admitted to the ICU. Detailed demographic characteristics of the nurses, physicians, and patients’ family members are presented in Tables 1-3, respectively. Four overarching themes were constructed through the reflexive analytical process, reflecting a logical progression from unmet clinical needs to technology adoption barriers for VR-based preoperative ICU visits. Among the 8 patients corresponding to family participants, surgical types included orthopedic trauma surgery, cholecystectomy for gallstones, gastrointestinal resection or anastomosis, partial hepatectomy, and vascular surgery; see Table 4 for details.

**Table 1.** Demographic characteristics of nurses (N=15).

Characteristic	Values
Age (y), mean (SD)	30.60 (7.97)
Gender, n (%)	
Man	7 (46.67)
Woman	8 (53.33)
Professional title, n (%)	
Nurse-in-charge	12 (80.00)
Senior nurse-in-charge	2 (13.33)
Chief nurse	1 (6.67)
Educational background, n (%)	
Bachelor’s degree	10 (66.67)
Master’s degree	4 (26.67)
Doctoral degree	1 (6.67)
Previous knowledge of VR^a^, n (%)	
Yes	13 (86.67)
No	2 (13.33)
Previous use of VR, n (%)	
Yes	3 (20.00)
No	12 (80.00)

a VR: virtual reality.

**Table 2.** Demographic characteristics of physicians (N=6).

Characteristic	Values
Age (y), mean (SD)	34.00 (7.72)
Gender, n (%)	
Man	2 (33.33)
Woman	4 (66.67)
Professional title, n (%)	
Attending physician	5 (83.33)
Chief physician	1 (16.67)
Educational background, n (%)	
Master’s degree	2 (33.33)
Doctoral degree	4 (66.67)
Previous knowledge of VR^a^, n (%)	
Yes	5 (83.33)
No	1 (16.67)
Previous use of VR, n (%)	
Yes	1 (16.67)
No	5 (83.33)

a VR: virtual reality.

**Table 3.** Demographic characteristics of patients’ family members (N=8).

Characteristic	Values
Age (y), mean (SD)	48.00 (13.93)
Gender, n (%)	
Man	4 (50.00)
Woman	4 (50.00)
Educational background, n (%)	
Primary school	1 (12.50)
Junior high school	1 (12.50)
Senior high school	2 (25.00)
University (bachelor’s)	3 (37.50)
Master’s degree	1 (12.50)
Occupation, n (%)	
Farmer	2 (25.00)
Worker	1 (12.50)
Staff (office worker)	2 (25.00)
Self-employed	1 (12.50)
Teacher	1 (12.50)
Retired	1 (12.50)
Previous knowledge of VR^a^, n (%)	
Yes	4 (50.00)
No	4 (50.00)
Previous use of VR, n (%)	
Yes	1 (12.50)
No	7 (87.50)

a VR: virtual reality.

**Table 4.** Clinical characteristics of patients associated with family participants (N=8).

Characteristic	Participants, n (%)
Type of surgery	
Orthopedic trauma surgery	2 (25.00)
Cholecystectomy for cholecystolithiasis	2 (25.00)
Gastrointestinal resection or anastomosis	2 (25.00)
Partial hepatectomy	1 (12.50)
Vascular surgery	1 (12.50)
Patient acuity	
Moderate severity	5 (62.50)
High severity	3 (37.50)

### Patients’ Family Members' Perceptions and Unmet Information Needs Regarding the ICU

#### Selective Knowledge Retention and Limited Information Access

Patients’ family members demonstrated distinct selective knowledge retention: they showed accurate understanding of surgical procedures and associated risks but had severe gaps in knowledge about the postoperative ICU stay. All patients’ family members reported relying primarily on nonprofessional sources (social media, anecdotal experiences from acquaintances) for ICU-related information; none received formal preoperative education about the ICU from health care providers.

*The ward doctors explained the surgery and its risks very clearly, but I have no idea about key questions like “What does the ICU look like?” “How does it operate?” or “How are emergencies handled if they occur?” I heard from the internet or news that only critically ill patients go to the ICU, but we can’t verify if this information is true or professionally sound.*

[Patients’ family member 1, man, 53 years old]

#### Prevalent Cognitive Biases and Stereotypes

Influenced by fragmented nonprofessional information, patients’ family members held three recurrent cognitive biases about the ICU: (1) environmental bias (perceiving the ICU as a closed, depressing space with limited family contact); (2) functional bias (equating the ICU exclusively with emergency rescue and end-of-life care); and (3) prognostic bias (believing ICU admission indicates a life-threatening condition with poor outcomes). Participants who held these cognitive biases tended to report greater preoperative anxiety.

*I think the ICU is a place where family visits are not allowed—closed and depressing.*

[Patients’ family member 4, man, 62 years old]

*When the doctor said my mother would be transferred to the ICU after surgery, I felt scared. My first thought was, “Are they going to perform an emergency rescue?”*

[Patients’ family member 5, woman, 40 years old]

#### Comprehensive and Detailed Information Needs

Patients’ family members’ information needs covered the entire process of the patient’s diagnosis, treatment, and care. These needs extended beyond core medical information to include care details and the work status of health care providers, reflecting a comprehensive and detail-oriented nature.

*Besides my family member’s condition in the ICU, I also want to see how the ICU staff work. If they are in good shape and their operations are professional, I feel much more at ease.*

[Patients’ family member 3, woman, 38 years old]

*What I care most about is the patient’s condition—his progress, treatment plan, and the functions of bedside equipment like monitors and ventilators.*

[Patients’ family member 8, woman, 26 years old]

### Willingness and Attitudes Toward VR-Based Preoperative ICU Visits

#### Universal Recognition of VR-Based Preoperative ICU Visits Necessity

All participants acknowledged the necessity of dedicated VR-based preoperative ICU visits, identifying three core benefits: (1) improved health education efficiency and knowledge retention, (2) reduced patient and family anxiety, and (3) potential reduction in health care provider workload by minimizing repetitive postoperative explanations.

*If patients know what to expect in the ICU before surgery, they won’t panic when they wake up. This will save us so much time explaining the same things repeatedly postoperatively.*

[Nurse 7, woman, 34 years old]

#### Anticipated Acceptance Driven by Perceived Usefulness

Most participants reported having heard of VR technology but lacked understanding of its specific functions and application scenarios. After receiving a detailed explanation of the VR concept and its potential clinical applications, all participants expressed positive willingness to use or recommend VR-based preoperative ICU visits. Acceptance was primarily driven by VR’s unique ability to create immersive visual experiences that address the inherent limitations of verbal education.

*This is an innovative form of visit—much more engaging than traditional verbal explanations. I’m very interested in this VR approach.*

[Patients’ family member 5, woman, 40 years old]

### User-Centered Design Requirements for VR Systems

#### Environmental and Procedural Simulation Requirements

All participants emphasized that the VR system must accurately simulate the ICU environment and postoperative procedures. Health care providers focused on realistic depiction of the ICU layout and bedside equipment, simulation of common clinical sounds (monitor alarms, ventilator noise), and explanation of typical postoperative sensations (eg, endotracheal intubation, physical restraints). Patients’ family members additionally requested a clear presentation of the full ICU admission workflow, staff roles, and expected discharge timing.

*Introduce the whole process of ICU stay, the roles and functions of medical staff, and when patients can be transferred out.*

[Patients’ family member 2, woman, 46 years old]

*We should not only show the ICU room configuration but also clarify what equipment (eg, monitors, ventilators) looks like. We could even simulate the alarm sounds of the equipment and the experiences patients may have in the ICU—including the function of endotracheal tubes, potential discomfort, physical restraints, and the responsibilities of doctors, nurses, and nursing assistants. Letting patients familiarize themselves with these “things they may see or hear” in advance will prevent them from panicking postoperatively.*

[Nurse 9, man, 38 years old]

#### Personalization and Accessibility Requirements

Health care providers stated that content should be personalized to patient characteristics (age, education level, surgery type) and stressed that a simple, intuitive interface is necessary to accommodate older adults and users with limited technology experience.

*It shouldn’t be too complicated, and there shouldn’t be too many operation steps. Otherwise, users—especially the elderly—will find it difficult and unwilling to use it.*

[Physician 2, man, 25 years old]

Patients’ family members shared the demand for ease of use, prioritizing concise core content, self-operation accessibility, and physical comfort.

*There is no need for too much content; just clearly explain the ICU environment and key procedures, preferably with simple and understandable user guidance.*

[Patients’ family member 6, man, 70 years old]

#### Practical Implementation Requirements

Health care providers, particularly frontline nurses, highlighted that the system must integrate seamlessly into existing clinical workflows, with minimal staff training and maintenance burden.

*When designing the VR system, we must prioritize simple operation. It must not be complicated—if it is difficult to operate, people will be unwilling to use it, no matter how good the system is. It should not increase the burden on clinical nurses.*

[Physician 3, woman, 36 years old]

Patients’ family members also expressed consideration for clinical implementation feasibility, stating that they did not want VR preoperative visits to disrupt routine ICU work or add to staff workload.

*ICU doctors and nurses are very busy. We would feel bad if conducting VR visits delayed their normal work.*

[Patients’ family member 2, man, 46 years old]

### Anticipated Implementation Barriers

#### Human Resource Constraints

All participating nurses identified insufficient human resources as the primary barrier to implementing VR-based preoperative ICU visits. They expressed concern that additional tasks related to VR system operation, patient guidance, and troubleshooting would increase their already heavy workload.

*ICU nurses already have a heavy workload. If we also have to take time to explain how to use VR and accompany patients through the visit, this will be time-consuming, and we will face difficulties in terms of staffing.*

[Nurse 6, woman, 24 years old]

To address this concern, health care providers proposed an interdepartmental collaboration strategy, reducing the exclusive workload burden on ICU staff through cross-ward coordination.

*We can cooperate with general wards and develop specialty tailored ICU VR visit content for different specialty wards to lighten the burden on ICU nurses.*

[Nurse 12, woman, 37 years old]

#### Technical and User-Related Barriers

Both health care providers and patients’ family members identified 2 common core barriers: VR-induced physical discomfort (eg, dizziness, nausea) and uneven technology acceptance across age and education groups, which may cause operational difficulties and poor content understanding among vulnerable users.

*Some patients will definitely experience motion sickness from 3D visuals, so they may not be able to use the system.*

[Nurse 1, woman, 24 years old]

*Rural patients or those with low educational levels may accept this new VR technology more slowly and have difficulty understanding it.*

[Nurse 10, man, 27 years old]

From the user perspective, patients’ family members proposed targeted strategies to improve acceptability, including on-site guidance for first-time use, simplified operation processes, and alternative access formats for those intolerant to head-mounted devices.

*If a nurse guides me the first time, I’d be willing to try it; otherwise, I wouldn’t know how to operate it.*

[Patients’ family member 7, man, 49 years old]

*I felt dizzy when I tried a VR headset before. For people who can’t tolerate wearing the device, I think the same content in a regular video form would also work.*

[Patients’ family member 5, woman, 40 years old]

## Discussion

### Summary of Main Findings

This qualitative study directly addresses our 2 primary research objectives: exploring stakeholder willingness and needs for VR-based preoperative ICU visits and identifying core design requirements and implementation barriers. Four overarching themes were constructed: (1) patients’ family members’ perceptions and unmet information needs regarding the ICU, (2) willingness and attitudes toward VR-based preoperative ICU visits, (3) user-centered design requirements for VR systems, and (4) anticipated implementation barriers. All participants held positive attitudes toward VR-based preoperative ICU visits, though their specific needs and priorities differed between health care providers and patients’ family members. Across all groups, 2 core tensions emerged that cut across these individual themes: first, a tension between families’ comprehensive information needs and health care providers’ imperative for workflow efficiency, and second, a tension between participants’ optimistic expectations based on limited VR experience and the practical challenges of real-world use.

### Detailed Discussion of Findings

#### Perioperative Information Asymmetry: A Persistent Clinical Gap

The most significant finding of this study is the profound perioperative information asymmetry experienced by patients’ family members of ICU-bound patients. While surgical consent processes ensure detailed communication about procedural risks, there is a near-complete absence of formal preoperative education about the ICU environment and postoperative care processes. This gap is associated with pervasive cognitive biases and higher self-reported preoperative anxiety among patients’ family members. This aligns with previous research linking inadequate preoperative ICU information to increased family distress and poorer patient outcomes [18].

Notably, patients’ family members in this study relied almost exclusively on nonprofessional information sources, which often present a distorted and fear-inducing view of the ICU. Crucially, this reliance did not reflect families’ failure to seek information—all participants actively sought ICU-related information—but rather, a systematic omission in existing care pathways. This distinction matters: the problem is not one of passive knowledge deficits but of active information seeking met with inadequate formal sources. Interpretively, families construct the ICU as an unfamiliar, closed, and threatening space. This finding highlights a notable unmet need in current perioperative care, which prioritizes medical information over psychosocial support. Given participants’ preference for visual and immersive information delivery, VR technology holds potential to address this gap by providing standardized, accessible ICU information.

#### Anticipated Acceptance of VR: Promise and Caveats

The near-universal anticipated acceptance of VR-based preoperative ICU visits observed in this study is particularly encouraging. This finding aligns with recent research demonstrating favorable attitudes toward VR-based interventions in preoperative education [19]. However, it is important to interpret these findings cautiously, as participants were evaluating a hypothetical intervention rather than an actual working system. Participants’ limited prior VR experience (7/8, 87.5%, of the patients’ family members had never used VR) may have contributed to their optimistic expectations. Previous research has shown that perceived acceptance of new technologies often differs from actual usability once users gain hands-on experience [20]. Therefore, while these findings provide a strong foundation for further development, they should be confirmed through subsequent usability testing with a prototype VR system.

#### User-Centered Design: Balancing Clinical Needs and Technical Feasibility

The design requirements identified in this study reflect a clear prioritization of clinical relevance and accessibility over technological sophistication. Both health care providers and patients’ family members emphasized the need for simple, intuitive operation and content tailored to diverse user populations. This aligns with a comprehensive digital health implementation framework that identifies user-centered design as the single most critical success factor for digital health technologies [21]. Notably, there was strong alignment between health care providers and patients’ family members on core design requirements, including accurate environmental simulation and clear explanation of postoperative sensations. At a deeper level, these needs show that care preparation is not only information delivery but also a process of reducing uncertainty and rebuilding a sense of control. This consensus simplifies the development process and increases the likelihood that the final system will meet the needs of all stakeholders. However, there were also important differences: patients’ family members prioritized comprehensive information about care processes, while health care providers emphasized workflow integration and minimal staff burden.

These differences point to a fundamental design tension that cannot be resolved through technical sophistication alone. Previous VR education studies have focused primarily on content accuracy and immersive quality, implicitly assuming that better simulation leads to better outcomes [22,23]. Our findings suggest this assumption is insufficient in the ICU context: the most immersive simulation is unlikely to be adopted if its operation disrupts nursing workflows or requires staff time that simply does not exist. Conversely, an overly simplified system that fails to address families’ comprehensive information needs may do little to build the realistic expectations that families actually seek. For VR designers, this means prioritizing operational simplicity not as a secondary usability consideration but as a core design requirement equal to content fidelity.

Based on these findings, we put forward a conceptual framework for the upcoming VR preoperative visit system. It will simulate the full ICU environment, medical devices and alarm sounds, and cover the whole process from admission preparation, in-hospital care to post-ICU transfer. The planned interaction modes include head-mounted devices, multiview switching, graphic displays, and voice guidance. Soft interface tones, on-site guidance, and family accompaniment are also incorporated to support future clinical use. Developed with users’ varied abilities in mind, this modular framework avoids an all-or-none experience and reduces information overload. Desktop VR is also recommended as an alternative for users who cannot use head-mounted displays, despite its weaker immersion.

#### Implementation Barriers: The Critical Role of Workflow Integration and End User Acceptance

Consistent with previous research, insufficient human resources were identified as the primary barrier to implementing VR-based preoperative ICU visits [24]. Frontline ICU nurses expressed significant concern that additional tasks related to VR system operation would increase their already heavy workload. This finding underscores the importance of implementation science in translating technological innovation into clinical practice. This barrier is not unique to critical care. A study of 23 rehabilitation health care professionals also identified staffing constraints and training burden as the most significant obstacles to VR adoption. This research further demonstrated that these challenges are disproportionately felt by professionals without prior VR experience, who require 2 to 3 times more training time than experienced users [25].

Our data suggest a specific nuance to this pattern: the concern about human resources was most pronounced among nurses with no prior VR experience, while VR-experienced nurses were more likely to see the technology as potentially time-saving in the long run. This implies that implementation strategies must address not only the objective time required but also the subjective perception of burden, which is shaped by familiarity and confidence with the technology. Successful adoption of VR-based preoperative ICU visits will require more than just a well-designed technology; it will require strategies to address systemic barriers, such as staffing constraints. Potential solutions include interdepartmental collaboration, dedicated technology support staff, or task shifting to nonclinical personnel. Several nurses in our study explicitly proposed interdepartmental collaboration as a practical solution, suggesting that implementation strategies should be designed collaboratively rather than top-down. Interventions that fail to address these practical considerations are unlikely to be sustained in the high-pressure, resource-constrained ICU environment.

Beyond clinical workflow considerations, end user acceptance and buy-in from patients’ family members are also prerequisites for successful implementation. In this study, patients’ family members broadly expressed willingness to use VR preoperative visits, providing a favorable foundation for rollout. However, user-side barriers identified in the interviews, including VR-induced physical discomfort (eg, motion sickness), operational difficulties for older or less digitally literate users, and uneven technology acceptance, may reduce real-world adherence if left unaddressed.

Consistent with previous research findings [26], motion sickness represents a common tolerability issue for head-mounted VR devices. However, this risk can be mitigated by controlling VR session duration [27,28] and optimizing device performance [29]. Importantly, our findings suggest that alternative access formats, such as regular video versions for users who cannot tolerate head-mounted displays, may be as important for ensuring equitable access as technical mitigation. To support user uptake, patients’ family members proposed targeted implementation strategies including on-site guidance for first-time use, simplified operation workflows, and alternative delivery formats. These user-generated solutions address real-world use scenarios and should be integrated into implementation plans alongside clinical workflow adjustments to ensure the intervention is both feasible for staff and accessible for end users.

### Strengths and Limitations

This study has several strengths, including the first dual perspective investigation of VR-based preoperative ICU visits in the Chinese context, purposive sampling achieving diversity across stakeholder groups, and rigorous reflexive thematic analysis with independent double coding.

However, several limitations warrant acknowledgment. First, this phase of the study did not include patient perspectives, which represents a significant limitation. This work constitutes the first phase of a multiphase user-centered design project for a VR preoperative visit system. Patients’ family members are core users of the VR system alongside patients, and they also provide essential emotional and informational support to patients during their ICU stay. Meanwhile, health care providers are key stakeholders in implementing any clinical intervention, and their input is critical to ensuring that the system can be integrated into existing clinical workflows. While the perspectives of patients’ family members and health care providers provide essential foundational information for designing a clinically relevant and implementable tool, we acknowledge that the absence of direct patient data limits the completeness of our conceptual framework. Patients are the ultimate end users of the system, and their experiences, preferences, and needs are essential for creating a truly comprehensive patient-centered intervention. Therefore, the design recommendations presented in this paper should be considered preliminary. Patient interviews have already been completed, and the manuscript presenting patient perspectives is currently in preparation. It will be submitted separately to complement the findings of this study, and the insights from the patient study will be used to refine and validate the current conceptual framework, completing the full user-centered design process.

Second, this was a single-center study conducted at a tertiary hospital in eastern China, which limits the generalizability of our findings. Health care systems and cultural contexts vary significantly across regions, and future multicenter studies are needed to confirm our results in different settings.

Third, the uniformly positive attitudes toward VR should be interpreted cautiously. Most participants, particularly patients’ family members, had limited hands-on VR experience, which may have led to overly optimistic perceptions. Additionally, the interview process may have introduced social desirability bias. These findings, therefore, represent preliminary expectations rather than confirmed real-world effectiveness. Limited prior VR experience among participants may have affected their ability to evaluate the technology realistically. As documented in previous research, individuals without hands-on VR experience often have difficulty accurately assessing its practical benefits and challenges. Their perceptions may change significantly once they have the opportunity to use an actual working VR system [25]. Future studies should evaluate the usability and effectiveness of a prototype VR system with actual users.

Fourth, the relatively small number (n=8) of patients’ family members may raise concerns about the comprehensiveness of our findings. However, it is important to note that the rigor of this qualitative study is evaluated by the depth of interpretive meaning and the adequacy of thematic development, rather than by statistical sample size [17]. While sufficient interpretive depth was achieved within this group, a larger sample could provide more diverse perspectives on family caregiver needs.

Fifth, social desirability bias may have influenced participants’ responses. Participants may have been more likely to express positive attitudes toward the technology to please the researchers. We attempted to minimize this bias by emphasizing that there were no right or wrong answers and that all opinions were valuable.

Sixth, this study relied solely on semistructured interviews and did not include methodological triangulation with observations or usability testing, which could have enhanced the validity of our findings.

Despite these limitations, this study provides critical insights into stakeholder perspectives on VR-based preoperative ICU visits, laying a solid foundation for system development and supporting further evaluation of its real-world feasibility and effectiveness.

### Conclusions and Broader Implications

This study identifies a critical gap in perioperative care: patients’ family members receive detailed information about surgical risks but almost no formal education about the ICU environment or postoperative care processes. Across all stakeholder groups, participants expressed positive anticipated acceptance of VR-based preoperative visits, recognizing their potential to address this gap through immersive, standardized information delivery.

These findings suggest that effective VR design must reconcile 2 potentially competing priorities: comprehensive environmental and procedural simulation that meets families’ information needs, and seamless integration into existing ICU workflows without adding to staff burden. The former underpins acceptance; the latter determines sustainability.

The immediate next step is prototype development followed by usability testing with actual end users, as the optimistic expectations expressed by participants with limited prior VR experience may not persist with hands-on use. If validated, VR-based preoperative ICU visits could offer a scalable solution to a persistent communication challenge in perioperative care.

## Abbreviations

COREQ: Consolidated Criteria for Reporting Qualitative Research
ICU: intensive care unit
VR: virtual reality

## References

[1] Wei G, Tan J, Ma F (2024). Barriers and facilitators of the nurse providing evidence-based preoperative visit-care for transcatheter aortic valve replacement: a mixed-methods study based on an evidence application setting. BMC Health Serv Res.

[2] Chiu PL, Li H, Yap KYL, Lam KMC, Yip PLR, Wong CL (2023). Virtual reality–based intervention to reduce preoperative anxiety in adults undergoing elective surgery: a randomized clinical trial. JAMA Netw Open.

[3] Rizzo MG, Costello JP, Luxenburg D, Cohen JL, Alberti N, Kaplan LD (2023). Augmented reality for perioperative anxiety in patients undergoing surgery: a randomized clinical trial. JAMA Netw Open.

[4] Xing J, Gong C, Wu B (2023). Effect of an educational video about ERAS on reducing preoperative anxiety and promoting recovery. Heliyon.

[5] Palanica A, Docktor MJ, Lee A, Fossat Y (2019). Using mobile virtual reality to enhance medical comprehension and satisfaction in patients and their families. Perspect Med Educ.

[6] Vlake JH, van Bommel J, Wils EJ (2021). Virtual reality for relatives of ICU patients to improve psychological sequelae: study protocol for a multicentre, randomised controlled trial. BMJ Open.

[7] Diseroad ER, Minnick S, Hutson TK (2024). Assessment of intensive care unit delirium in developmentally delayed children. Pediatr Res.

[8] Bruno RR, Wolff G, Wernly B (2022). Virtual and augmented reality in critical care medicine: the patient’s, clinician’s, and researcher’s perspective. Crit Care.

[9] Li C, Yu H, Wen J, Li L (2025). Influence of IDEAS preoperative visit mode on postoperative rehabilitation of patients undergoing laparoscopic cholecystectomy: a randomized clinical trial. J Perianesth Nurs.

[10] Guo X, Qi K, Wu H (2025). The effect of nurse-led preoperative visits on anxiety: an integrative review. J Perianesth Nurs.

[11] van der Kruk SR, Zielinski R, MacDougall H, Hughes-Barton D, Gunn KM (2022). Virtual reality as a patient education tool in healthcare: a scoping review. Patient Educ Couns.

[12] Yang J, Rhu J, Lim S (2024). Impact of virtual reality education on disease-specific knowledge and anxiety for hepatocellular carcinoma patient scheduled for liver resection: a randomized controlled study. Int J Surg.

[13] Lee J, Ryu JH, Kim JH, Han SH, Park JW (2025). Effects of a virtual reality digital twin of the operating theatre on anxiety in pediatric surgery patients: a randomized controlled trial. Korean J Anesthesiol.

[14] Palinkas LA, Horwitz SM, Green CA, Wisdom JP, Duan N, Hoagwood K (2015). Purposeful sampling for qualitative data collection and analysis in mixed method implementation research. Adm Policy Ment Health.

[15] Tong A, Sainsbury P, Craig J (2007). Consolidated Criteria for Reporting Qualitative Research (COREQ): a 32-item checklist for interviews and focus groups. Int J Qual Health Care.

[16] Braun V, Clarke V (2006). Using thematic analysis in psychology. Qual Res Psychol.

[17] Kang R, Xuan Z, Tong L, Wang Y, Jin S, Xiao Q (2025). Nurse researchers’ experiences and perceptions of generative AI: qualitative semistructured interview study. J Med Internet Res.

[18] Lai VKW, Ho KM, Wong WT (2021). Effect of preoperative education and ICU tour on patient and family satisfaction and anxiety in the intensive care unit after elective cardiac surgery: a randomised controlled trial. BMJ Qual Saf.

[19] Yu Y, Zhou X, Zeng G, Hou Y (2023). Impact of virtual operating room tours on relieving perioperative anxiety in adult patients: a systematic review. J Perianesth Nurs.

[20] Ferrer Costa J, Armayones Ruiz M (2026). Enhancing the predictive value of formative evaluation in extended reality adoption: addressing the experience gap. JMIR Form Res.

[21] Loo RTJ, Nasta F, Macchi M (2025). Recommendations for successful development and implementation of digital health technology tools. J Med Internet Res.

[22] Samsudin A, Zahran M, Nugraha E (2026). Immersion is not enough: design quality as the key determinant of perceived effectiveness and usage intention in educational virtual reality. Comput Educ X Reality.

[23] Wenk N, Penalver-Andres J, Buetler KA, Nef T, Müri RM, Marchal-Crespo L (2023). Effect of immersive visualization technologies on cognitive load, motivation, usability, and embodiment. Virtual Real.

[24] Kouijzer MMTE, Kip H, Bouman YHA, Kelders SM (2023). Implementation of virtual reality in healthcare: a scoping review on the implementation process of virtual reality in various healthcare settings. Implement Sci Commun.

[25] Schreiter M, Hennrich J, Wolf AL, Eymann T (2025). The influence of previous experience on virtual reality adoption in medical rehabilitation and overcoming knowledge gaps among health care professionals: qualitative interview study. J Med Internet Res.

[26] Saab MM, Hegarty J, Murphy D, Landers M (2021). Incorporating virtual reality in nurse education: a qualitative study of nursing students’ perspectives. Nurse Educ Today.

[27] Gao J, Liu S, Zhang S (2022). Pilot study of a virtual reality educational intervention for radiotherapy patients prior to initiating treatment. J Cancer Educ.

[28] Aardoom JJ, Hilt AD, Woudenberg T, Chavannes NH, Atsma DE (2022). A preoperative virtual reality app for patients scheduled for cardiac catheterization: pre-post questionnaire study examining feasibility, usability, and acceptability. JMIR Cardio.

[29] Cipresso P, Giglioli IAC, Raya MA, Riva G (2018). The past, present, and future of virtual and augmented reality research: a network and cluster analysis of the literature. Front Psychol.

